# Cling together, swing together? Assessing indirect retrieval of stimulus-response bindings for associated stimuli

**DOI:** 10.3758/s13423-024-02525-0

**Published:** 2024-06-14

**Authors:** Mrudula Arunkumar, Klaus Rothermund, Wilfried Kunde, Viola Mocke, Carina G. Giesen

**Affiliations:** 1 Department of General Psychology II, Institute for Psychology, Friedrich-Schiller University Jena, Am Steiger 3/Haus 1, 07743 Jena, Germany; 2 Department of Psychology III, Julius-Maximilians-University Würzburg, Würzburg, Germany; 3 Department of Psychology, Faculty of Health, Health and Medical University Erfurt, Am Anger, 64-73, 99084 Erfurt, Germany

**Keywords:** Stimulus-response bindings, Episodic retrieval, Event files, Stimulus-stimulus associations, Association formation

## Abstract

When a stimulus is paired with a response, a stimulus-response (SR) binding (or event file) is formed. Subsequent stimulus repetition retrieves the SR binding from memory, which facilitates (impedes) performance when the same (a different) response is required. We aimed to explore whether *indirect* retrieval of SR bindings by a newly learnt associated stimulus is possible. Participants first went through a learning task to acquire *novel* stimulus-stimulus associations. The same stimulus pairs were then presented in a prime-probe task to assess direct and indirect retrieval effects. Participants responded by classifying word color in prime and probe trials. Probe words were either identical to prime words (test for direct retrieval) or corresponded to the associated stimulus (test for indirect retrieval) or were unrelated words (baseline). Independently of word relation, response relation (repetition vs. change) across prime and probe trials was manipulated. In two highly powered preregistered studies (total *N* = 260) using different types of stimulus associations, we obtained evidence for direct retrieval due to identical word repetition in the probe. Crucially, evidence for indirect retrieval upon presentation of an associated probe word was absent. Controlling for memory of each stimulus-stimulus association did not alter the findings. Our results show that indirect retrieval through newly acquired associations does not occur at the level of SR bindings, at least not for recently acquired stimulus-stimulus associations. Our study illustrates the scope of binding principles and highlights boundary conditions for the stimulus properties that can elicit automatic response retrieval.

## Introduction

When you respond to a particular stimulus, this is encoded as a memory episode, termed stimulus-response (SR) binding or *event file* (Hommel, 1998). When the stimulus repeats, the previous SR binding is retrieved from memory, which affects your performance (Rothermund et al., 2005). If the retrieved SR binding is appropriate, performance typically benefits from the retrieval of SR bindings. Contrarily, if the retrieved SR binding does not match the currently required response, performance costs accrue. These behavioral phenomena show the extent to which SR bindings influence our actions and performance. Notably, even task-irrelevant stimuli that were presented along with the target stimuli can later retrieve the respective response (Frings et al., 2007). Generally, SR binding and retrieval (SRBR) effects are documented for various stimuli and responses, which attests that binding and retrieval are core mechanisms of action regulation (Frings et al., 2020; Kiesel et al., 2023).

Several studies explored the scope of conditions that can trigger retrieval of SR bindings (Frings et al., 2013; Horner & Henson, 2011; Laub & Frings, 2020; Singh et al., 2016). Findings indicate that retrieval is not limited to the repetition of a perceptually identical stimulus (although this is by far the most potent condition to trigger retrieval processes; Hommel, 2005). Singh and colleagues (Singh et al., 2016; Schöpper et al., 2020) varied stimulus similarity via the luminance of irrelevant shapes. Even for perceptually similar, yet non-identical stimuli, they obtained SRBR effects, which were diminished with increasing stimulus dissimilarity. However, here, perceptual similarity coincided with semantic overlap. Resolving this caveat, Laub and Frings (2020) provided evidence for retrieval of SR bindings that is due to perceptual similarities even in conditions where semantic meaning clearly differs between integration (i.e., creation of bindings) and later retrieval. Hence, retrieval can also be triggered by different stimuli that are perceptually similar and semantically dissimilar (e.g., *star* retrieves bindings with *scar*; Laub & Frings, 2020).

This research is complemented by evidence for SRBR effects for perceptually dissimilar but semantically related stimuli (Frings et al., 2013; Horner & Henson, 2011). For instance, presenting the *picture* of a frog retrieved bindings between the *sound* of a frog and a previous response (or vice versa; Frings et al., 2013). This latter finding is of particular importance for our study, because it shows that retrieval effects can be conceptually mediated and can thus occur between *different* distractor stimuli that are linked via an overlearned association that is represented as a common concept. Importantly, activation of the concept does not depend on particular identical stimuli, but can result from activation by different stimuli (e.g., the concept “frog” can be activated by presenting pictures of a frog, hearing the sound of a frog, etc.). Overlearned associations are the result of a learning history of many past pairings of different stimuli sharing the same semantic meaning. These overlearned associations are typically part of semantic long-term memory that is easily accessible for automatic retrieval (Marron et al., 2020; see also Kumar et al., 2021). However, one may wonder whether it actually takes that many encounters with different stimuli (e.g., actual exposures to frogs and their sounds) to form an overlearned association between both types of stimulation. Possibly, rather few trials are already sufficient to create a common connection between two stimuli. Even though retrieval can be triggered by other associated conceptual representations (Frings et al., 2013, Horner & Henson, 2011), it is yet to be explored whether automatic retrieval can be triggered via newly learnt stimulus associations. In the learning literature, a form of this is indicated in sensory preconditioning where newly established stimuli associations can indirectly activate a response that was only learnt with the associated stimulus (Brogden, 1939).

In the present study, we explore whether a stimulus can retrieve a response that was given to another, previously associated, stimulus. Thus, we studied indirect response retrieval that is mediated via stimulus-stimulus (S-S) associations that are newly learnt. Contrary to existing studies, we are not interested in overlearned associations or pre-existing concepts (Frings et al., 2013). Instead, we focus on *newly acquired* S-S associations that are characterized by a very recent learning history. Our research is motivated by a recent study on contingency learning, showing that a stimulus can indirectly activate learned responses that were contingently paired with another associated stimulus during a training phase within the same experiment (Arunkumar et al., 2024). In the first phase, participants learnt different S-S associations. In a second phase, participants then learnt a stimulus-response contingency for one stimulus of each S-S pair (e.g., S1). In a critical test phase, it was assessed whether the *associated* stimulus (S2) can also activate the response that was previously contingently paired with S1. Indeed, this was the case, as participants were more likely to respond to S2 with the response that was previously associated with S1. On a conceptual level, the described method is reminiscent of the sensory preconditioning procedure known from Pavlovian conditioning (e.g., Barr et al., 2003; Brogden, 1939). This means that sensory preconditioning-like effects also emerge for arbitrary pairings between stimuli and voluntary responses in the contingency learning paradigm. As transient SR bindings are discussed as the cognitive basis of contingency learning effects (e.g., Giesen et al., 2020; Schmidt et al., 2020), one could speculate that indirect retrieval effects should not only emerge for learnt SR contingencies (as seen in Arunkumar et al., 2024), but could possibly also occur at the level of transient bindings. Note that transient SR bindings emerge non-contingently as a by-product of contiguous co-occurrence of stimuli and responses. Even though SRBR effects were found for perceptually dissimilar but conceptually related stimuli (Frings et al., 2013; Horner & Henson, 2011), the novelty of this study lies in exploring whether newly learnt associated stimuli can retrieve responses that were never directly linked with them in an episodic rather than contingency-based fashion, as in Arunkumar et al. (2024). This research further extends the knowledge regarding the scope of conditions that can trigger retrieval processes and to which extent this is mediated by past (recent or overlearned) associations.

## Method

### Experiments 1a and 1b

We investigated two types of S-S associations in two independent studies, one with name-trait visual word pairs (Experiment 1a) and the other with audiovisual word – pseudoword pairs that resemble a new language-learning scenario (Experiment 1b). Different S-S pairs were used in each experiment to explore the extent of indirect retrieval effects. In Experiment 1a, unimodal visual pairs that have a plausible connection were used in the form of name-trait pairs as these are commonly encountered while describing people. To further explore the indirect retrieval effects, multimodal pairs were used in Experiment 1b as they have shown stronger indirect response activation effects (Arunkumar et al., 2024). Moreover, these multimodal pairs were intended to resemble a language learning setup since we tend to pick up new words with both visual and audio input. Language learning literature has also shown that semantic properties from words can be transferred to other words or pseudowords (Staats et al., 1959). Hence in Experiment 1b, word-pseudoword pairs were learned and were then used to test indirect retrieval effects.

The general paradigm of both experiments was as follows: Participants learnt novel associations between stimuli in an *S-S association formation phase*. The same word stimuli were then presented in a prime-probe paradigm to assess direct and indirect retrieval of previous SR bindings. Probe words were either identical (test for direct retrieval) or associated (test for indirect retrieval) with previous prime words, or were unrelated words (baseline). The relation between prime and probe responses (repetition vs. change) was manipulated independently. This design allowed us to test whether presenting the associated stimulus can access and retrieve a SR binding from the previous prime trial. We hypothesized that SRBR effects (indicated by a Word Relation × Response Relation interaction) occur not only when the identical word appears, but also when the associated word appears in the probe. All materials, preregistrations, data, and analyses for both the experiments are publicly available on the Open Science Framework repository (OSF; https://osf.io/jpv8y/)

#### Openness and transparency

An a priori power analysis (G*Power 3.1; Faul et al., 2007) was done to determine the required sample size to detect an effect size of *d*_*z*_ = 0.22. This estimate was based on a pilot experiment^1^ that we planned to replicate with a higher sample size. Given the effect size from the pilot study, and a statistical power of 1−ß = 0.80 in one-tailed dependent-samples *t*-tests with α = 0.05, a sample size of *N* = 130 was required for Experiment 1a and Experiment 1b. The design and the analysis plan were preregistered (Experiment 1a: 10.17605/OSF.IO/W9GEH; Experiment 1b: 10.17605/OSF.IO/6WN93).

In accordance with the ethical standards at the Institute of Psychology at University Jena, no ethics approval was required because no cover-story or suggestive information was conveyed to participants and everyone received an extensive debriefing.

#### Participants

For Experiment 1a, *N* = 130 native English-speaking participants (M_age_ = 27.8 years, range: 18–35 years, 83 females) were recruited online via Prolific Academic (https:/prolific.co/). The experiment duration was 25 min. Participants received £3.75 for participation. Explicit informed consent was collected electronically at the beginning of the study. Note that we had preregistered to exclude participants who showed an accuracy score of 0 in the cued recall test for the S-S association phase at the end of the study. This applied to *N* = 22 participants in Experiment 1a. Since we felt that a stimulus-wise evaluation of S-S association strength is a better indicator of associative strength among S-S pairs than the person-centered approach, we decided against eliminating data based on participants’ overall accuracy score. We therefore kept these participants in the sample to avoid problems of low statistical power as this number was considerably higher than anticipated and focused on memory performance per stimulus as an additional predictor in our analyses.

For Experiment 1b, we directly preregistered stimulus-specific memory performance as an additional predictor. We recruited *N* = 130 native German speakers (M_age_ = 25.1 years, range: 18–35 years, 63 females) also via Prolific and they were compensated with £3.75. Only German native speakers were recruited because in Experiment 1b the stimuli consisted of German words. Informed consent was obtained at the beginning of both the experiments by a keypress upon reading the consent form containing details of the study.

#### Material and procedure

**Fig. 1:**
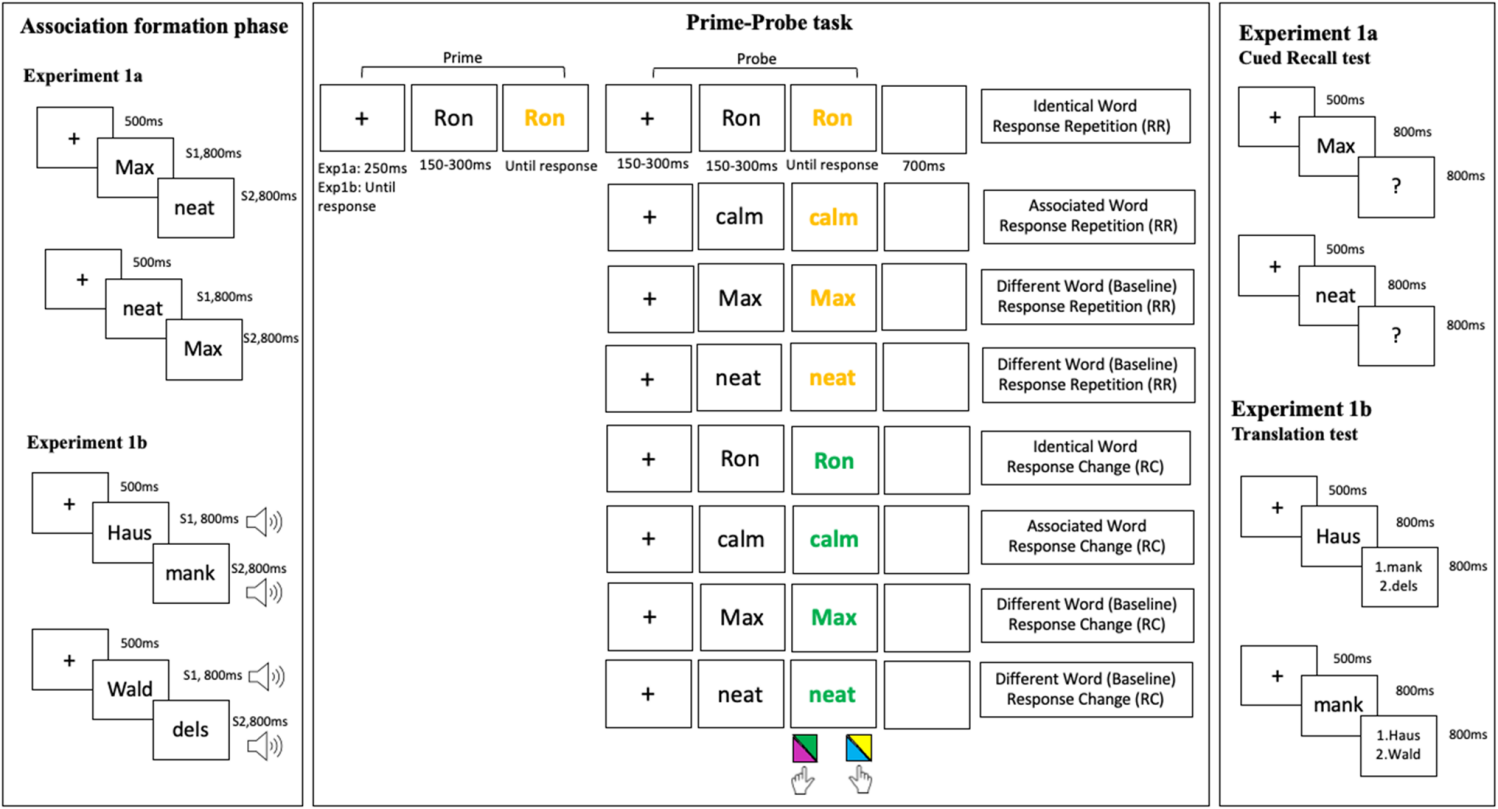
Illustration of experimental structure and example trial sequences in Experiment 1a. Experiment 1b followed a similar trial sequence with minor changes in the prime probe task (i.e., additional auditory stimulus presentation and trials only starting upon a press of the spacebar). Display colors are inverted for illustrative purposes; in both the experiments, words were displayed on a black background with white/colored font (see text for details). In prime trials, both S1 and S2 could appear

For both the experiments, the participants were instructed to only use their laptop. The study consisted of two parts: An association formation phase, followed by a prime-probe task (Fig. 1). Unless reported otherwise, all words were displayed in white Arial font sized 0.04% of the respective monitor’s height (using Psychopy, Peirce et al., 2019), on a black screen in the association phase.

In the association formation phase, two stimulus pairs (S1-S2) were introduced in the study and were presented 40 times each. In Experiment 1a, the stimulus pairs consisted of name-trait word pairs (e.g., *Ron-calm; Max-neat*), whereas in Experiment 1b, the stimulus pairs consisted of pairs of German words (*Haus* [house], *Wald* [forest]) and pseudowords (e.g., *mank, dels*). In Experiment 1b, the S-S pair mapping was counterbalanced across participants. Crucially, in Experiment 1b, the stimuli pairs were also presented auditorily in addition to the visual presentation of the word. For the second experiment we chose these stimuli and the number of presentations because in Arunkumar et al. (2024) where the same stimulus pairs were used (but German words were presented only visually and pseudowords were presented only auditorily), robust S-S associations in a contingency learning paradigm were established.

In both experiments, we asked participants to observe the presentation of the two words, which appeared in succession, and to say the words aloud. Participants were instructed that they had to say the word aloud as the voice responses were recorded. This was used to sustain participants’ focus throughout the association phase to help remember the S-S associations. However, we actually did not record their voices or use any input from their microphones. At the end of the study, we informed participants regarding this and told them that none of their voices were recorded or saved. Forty occurrences of each pair were presented out of which 20 occurrences were presented with the S1 first and 20 with the S2 appearing first, resulting in a total of 80 trials. This enabled bidirectional learning of the S-S associations. A trial in the association formation phase was as follows: A centrally presented fixation cross (500 ms) was followed by the S1 (e.g., *Max* in Experiment 1a or *Haus* in Experiment 1b) for 800 ms. Then, the S2 (e.g., *neat* in Experiment 1a or *mank* in Experiment 1b) was displayed for 800 ms, which was followed by the fixation cross for the next trial (Fig. 1). In Experiment 1b, the visual presentation was also accompanied by the auditory presentation of the word/pseudoword for 800 ms.

Then, the sequential prime-probe task followed. Participants classified the color of the word in prime and probe trials by pressing *D* for green or pink words and pressing *L* for blue or yellow words. Two colors were mapped onto each response key to avoid a confound between response repetition and color repetition (i.e., even in response repetition trials, stimulus color could change from prime to probe). Based on the word presented in the prime trial, the probe trial either displayed the exact same word (identical repetition, ID, 25%), the associated word (associated, AS, 25%), or a different word (baseline, B, 50%), which was one of the two words from the other stimulus pair. Half of all probe trials required the same response as the preceding prime trial (response repetition, RR), whereas the other half required a different response (response change, RC). The color in which the prime and probe words appeared was balanced (25% each of the four colors). Half of all RR sequences repeated the prime color in the probe (e.g., pink-pink), whereas the remaining RR sequences changed the color in the probe (e.g., pink-green). By definition, all the RC sequences presented probe words in a different color (assignment of colors to RC sequences was balanced, too, meaning that both colors assigned to a key were presented equally often in RC sequences). The prime-probe task consisted of 256 prime-probe trial sequences. In Experiment 1a, these prime-probe sequences began with a fixation cross (250 ms), followed by the prime trial in which a word appeared in white font (150–300 ms in 50 ms steps randomly chosen in every trial) to prevent anticipatory responses, which then changed to one of four target colors: green, yellow, blue, and pink (until response). Then, another fixation cross appeared (150–350 ms in 50 ms steps; *M* = 250 ms), followed by the probe trial: A word appeared in white font (150–300 ms in 50-ms steps; randomly chosen), which then changed to one of four colors (until response). After a blank black screen (700 ms), the next prime-probe sequence started (Fig. 1). Due to the audiovisual nature of the stimuli in Experiment 1b, the trial sequence was slightly modified from Experiment 1a, where the fixation cross was displayed until a spacebar was pressed to indicate the beginning of the current prime-probe sequence. Then the prime trial started with visual presentation of the stimulus in white font (150–300 ms in 50-ms steps randomly chosen in every trial) along with the auditory presentation of the stimulus. The visual stimulus then changed into one of four colors: green, yellow, blue and pink (until response). Then, another fixation cross appeared (150–350 ms in 50-ms steps; *M* = 250 ms), followed by the probe trial. Also in the probe trial, the visual presentation of the stimulus was first displayed in white (150–300ms in 50-ms steps randomly chosen in every trial) along with the auditory presentation of the stimulus. The visual stimulus then changed into one of four colors: green, yellow, blue and pink (until response). Similar to Experiment 1a, the prime-probe sequence ended with a blank black screen for 700 ms following which the next sequence began. In both experiments, there were two self-paced breaks during this phase.

At the end of the Experiment 1a, we presented a cued recall test to assess participants’ memory of the S-S associations. After a fixation cross (500 ms), each of the four words was presented (800 ms), followed by a “?” (800 ms). Following this, a screen appeared asking the participants to choose the word that should have appeared. Participants had to select the correct associated word from a list of options, including (1) the correct associated word, (2) a word from the other pair, and (3) a do not know option. The order of the options was randomly determined for every trial for each participant. Participants gave their response by pressing the corresponding numbers on their keyboard. In Experiment 1b, the cued recall test was replaced by a translation questionnaire, where participants were asked four questions one by one with a blank black screen as an inter-trial interval of 700 ms. Two questions asked what the German words translate to and the other two questions asked what the pseudowords mean, as the German word-pseudoword stimulus association that was built in the association phase resembled a language-learning scenario. Participants chose the response by pressing the corresponding number that displayed the options. (Fig. 1). The options were either the two German words or the two pseudowords depending on the question. The order of the response options was randomized for each trial.

#### Design and data analysis

Both the experiments had a 2 Response Relation (RR vs. RC) × 3 Word Relation (ID vs. AS vs. B) within-subjects design. Only reaction times (RTs) in the probe trials were analyzed as these were preregistered as the primary dependent measure, since they are more robust to detect SRBR effects.

We hypothesized that there would be an SRBR effect, reflected in a significant response relation by word relation interaction. In Experiment 1a, we specified two a priori orthogonal contrasts for the Word Relation factor to compare indirect retrieval for the associated word stimuli with direct retrieval for identical word repetitions. According to contrast 1, the two-way interaction (referring to the difference in the word relation effect between RR and RC conditions) should be significantly different from zero for both identical word repetitions and associated probe words, compared with baseline (contrast 1 1 -2). According to contrast 2, retrieval effects (i.e., differences between RR and RC conditions) should be of equal magnitude for identical word repetitions and associated probe words (contrast 1 -1 0, which should not differ from zero), thus expecting that indirect retrieval effects are comparable in size to direct retrieval effects. For Experiment 1b, we specified different a priori contrasts that were motivated by the findings of Experiment 1a and provided a direct measure of testing direct and indirect SRBR effects. The first contrast represents the direct SRBR effect by comparing the interaction for the identical probe words versus word change (1 0 -1) and the response relation factor. This contrast should be significantly different from zero (directional test) and should reflect standard SR binding and retrieval effects. The second contrast reflects the indirect retrieval effect by comparing whether there is also a significant interaction between associated probe word versus word changes (0 1 -1) and the response relation factor. If this test is significant, we can assume that indirect retrieval effects are present since the associated words are also exhibiting binding and retrieval effects like the identical stimulus relation condition. We used R (Version 4.2.1; R Core Team, 2021) to analyze the data and the packages *afex* and *emmeans* to perform the ANOVA and contrasts analysis.

## Experiment 1a: Results

### Response retrieval effects

After removing erroneous probes (4.5% of the trials), probes following erroneous primes (5.5% of the trials) as well as probe RT outliers^2^ (5% of the trials; leading to a total of 15% out of which 0.5% trials have both prime and probe errors, thus resulting in an overall exclusion: 14.5.% of all trials), mean probe RT was entered as a dependent variable to a 2 (Response Relation) × 3 (Word Relation) repeated-measures ANOVA.

**Fig. 2:**
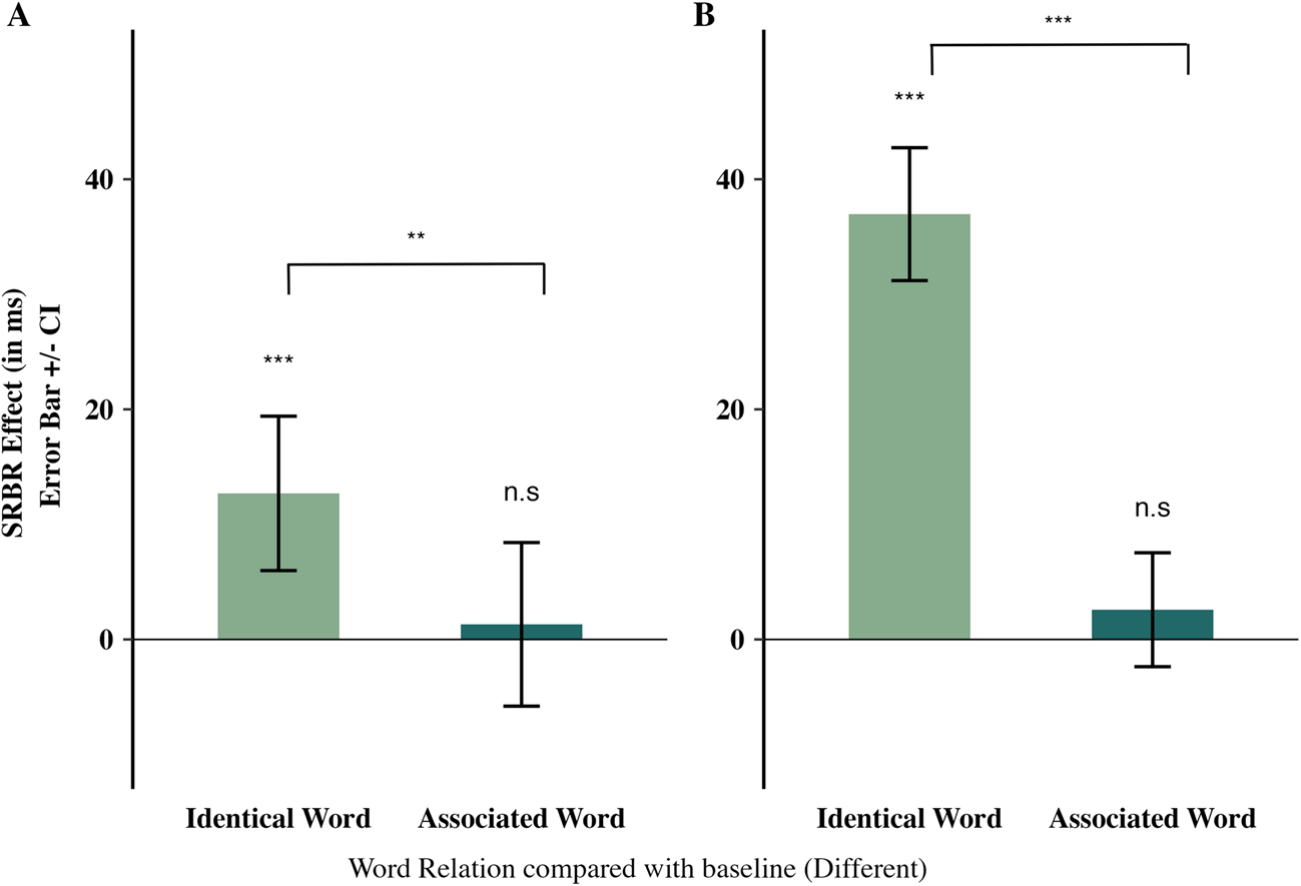
The stimulus-response binding and retrieval (SRBR) effects (i.e., effects of response relation) for identical probe words vs. associated probe words compared to the baseline (different probe word), respectively for Experiment 1a (**A**) and Experiment 1b (**B**) (see Table 1 for effect computation). *Note:* ** indicates *p* <. 05, *** indicates *p* < .005

The results showed a main effect of response relation, reflecting faster performance for RR (*M* = 546 ms) than for RC trials (*M* = 612 ms), *F*(1,129) = 217.08, *p* < .001, η_p_^2^ = 0.63, but no effect of word relation, *F* < 1. However, relevant to our hypothesis, we found a significant interaction, *F*(2,258) = 6.77, *p* < .001, η_p_^2^ = 0.05, indicating an SRBR effect. We further decomposed the interaction using the preregistered a priori contrasts. As predicted, Contrast 1 (1 1 -2 for the word relation levels ID, AS, and B) yielded a significant difference, *t*(129) = 2.56, *p* = .012, *d*_*z*_ = 0.22, whereas, against our predictions, Contrast 2 (1 -1 0 for the word relation levels ID, AS, and B) was significant, *t*(129) = 2.63, *p* = .010, *d*_*z*_ = 0.23. These findings suggest that retrieval effects differed between identical and associated words. We conducted additional post hoc analyses and computed SRBR effects separately for identical and associated word presentations (see Table 1 for details on effect computation, and Fig. 2). For identical word repetitions, robust SRBR effects emerged that significantly differed from zero, *t*(129) = 3.74, *p* < .001, *d*_*z*_ = 0.33 (note that the obtained effect size corresponds to the typical range of SRBR effects for irrelevant words; see Footnote 1), due to a significant performance benefit of Δ_(B-ID)_ = 7.2 ms, *t*(129) = 2.87, *p* < .005, *d*_*z*_ = 0.25, for RR sequences, and a significant performance cost of Δ_(B-ID)_ = -6 ms, *t*(129) = 2.60, *p* = .005, *d*_*z*_ = 0.22, for RC sequences. To supplement our frequentist analyses, as an exploratory measure we also computed Bayes factors using JASP (van Doorn et al., 2021; Rouder et al., 2009) for the *post hoc* contrasts, with the priors being described by a Cauchy distribution centered around 0 with a width parameter of 0.707 (default priors in JASP, v.0.18.1). We used a Bayesian one-sample *t*- test with the alternative hypothesis predicting the effect to be greater than zero which resulted in a BF_+0_ = 132, providing strong evidence for the alternative hypothesis according to van Doorn et al. (2021). For associated word presentations in the probe, SRBR effects were virtually absent and did not differ from zero, *t*(129) = 0.36, *p* = .358, *d*_*z*_ = 0.03. In a Bayesian one-sample* t*-test with the null hypothesis predicting that the effect is not greater than zero, we found a BF_0+_ = 7.5 indicating moderate evidence for the null hypothesis. These analyses show that the associated probe words did *not* retrieve the responses bound to their associated stimulus.

**Table 1:** Mean (SD) probe reaction times (in ms) for the factorial design

	Experiment 1a			Experiment 1b		
Word Relation Prime ➔ Probe	Response Relation Prime ➔ Probe		SRBR Effect^a^	Response Relation Prime ➔ Probe		SRBR Effect^a^
	Response Repetition (RR)	Response Change (RC)		Response Repetition (RR)	Response Change (RC)	
Identical word (ID)	541 (84)	616 (97)	12.7 (38.7)	473 (27)	552 (21)	36.9 (33.1)
Different word, baseline (B)	548 (81)	610 (99)		499 (19)	541 (20)	
Associated word (AS)	548 (85)	611 (95)	1.31 (40.9)	497 (21)	542 (23)	2.58 (28.4)

^a^SRBR Effect = Stimulus-response binding and retrieval effects. For the ID condition, SRBR effects are computed as SRBR_ID_ = (B − ID)_RR_ − (B − ID)_RC_. For the AS condition, SRBR effects are computed as SRBR_AS_ = (B − AS)_RR_ − (B − AS)_RC_. For SRBR effects, positive values indicate retrieval of SR bindings that is due to performance benefits for RR sequences (e.g., (B − ID)_RR_ > 0) and performance costs for RC sequences (e.g., (B − ID)_RC_ < 0), respectively

### Memory of S-S association

The memory of the S-S association was assessed using the performance in the cued recall test that was presented at the end of the experiment. For each of the four stimuli from both word pairs, participants were asked what the associated word would be. Mean accuracy rates per item across participants show that participants were able to accurately identify the associated adjective word significantly above chance for *Max* (58% correct responses), *t*(129) = 1.77, *p* = .039 (one-tailed), and *Ron* (61% correct responses), *t*(129) = 2.89, *p* = .002 (one-tailed). When the two adjective words were presented first, mean accuracy rates were lower and did not differ significantly from chance, neither for *neat* (55% correct responses), *t*(129) = 1.23, *p* =.110 (one-tailed), nor for *calm* (57% correct responses), *t*(129) = 1.59, *p* =.057 (one-tailed; see Table 2).

**Table 2:** Mean accuracy rate in the cued recall test per item in Experiment 1a and the translation task in Experiment 1b

	Test item/ option				Directional *t*-tests comparing accuracy rate with the correct option (highlighted in bold font), against 0.5 (chance)
Experiment 1a		Calm	neat	Do not know	
	Max	0.22	**0.58**	0.20	*t*(129)=1.77, *p* = .039
	Ron	**0.62**	0.20	0.18	*t*(129)=2.89, *p =* .002
		Max	Ron	Do not know	
	calm	0.25	**0.57**	0.18	*t*(129) = 1.59, *p* = .057
	neat	**0.55**	0.35	0.19	*t*(129) = 1.23, *p =*.110
Experiment 1b		Associated pseudoword			
	Haus	**0.81**			*t*(128) = 9.18, *p* <.001
	Wald	**0.78**			*t*(128) = 7.77, *p* <.001
		Associated German word			
	mank	**0.77**			*t*(128) = 7.16, *p* <.001
	dels	**0.85**			*t*(128) = 10.78, *p* <.001

To investigate how item-specific memory contributes to indirect retrieval effects for associated words, a post hoc multi-level analysis on probe trial RT was computed. In detail, we ran a linear mixed-effects model with random intercepts with trial-based predictors as level 1 variables and participants as level 2 predictors using *lmer* in R and included probe trial RT as dependent variable. We added fixed effects for word relation (only two levels were considered and contrast coded: associated = 0.66 vs. baseline = -0.33), response relation (contrast coded: RR = 0.5, RC = -0.5), and item-specific accuracy in the cued-recall test for associated words (which was contrast coded: accurate = 0.42, inaccurate = -0.58) and their interactions. Participants were added as a random effect. The results are presented in Table 3. Most importantly, this analysis did not yield a significant three-way interaction between item-specific S-S recall accuracy, word relation, and response relation (*p* = .493; Table 3). Put differently, whether or not the specific participant responded to a word (e.g., *Max*) with the correct associated word (e.g., *neat*) in the cued recall test, did not modulate the strength of the respective indirect prime-response retrieval effects. This further illustrates that even the ability to remember a specific S-S association did not moderate the indirect retrieval effects for the associated word.^3^

**Table 3:** Multi-level model with word relation (associated probe word vs. baseline), response relation (repetition vs. change), and of S-S association recall per item (correct vs. incorrect) and their interactions for both Experiment 1a and Experiment 1b

	Experiment 1a				Experiment 1b			
Effects	Β	SE	*t* statistic	*p*	β	SE	*t* statistic	*p*
Intercept	579.49	7.50	77.27	<.001	519.63	5.90	88.04	<.001
Word Relation (associated vs. baseline)	0.96	1.80	0.53	.595	-0.07	1.40	-0.05	.959
Response Relation (repetition vs. change)	62.22	1.67	37.15	<.001	42.33	1.32	31.98	<.001
S-S recall (correct vs. incorrect)	1.35	2.29	0.59	.556	-0.68	2.70	-0.25	.802
Word Relation * Response Relation	0.74	3.59	0.21	.836	2.51	2.81	0.80	.371
Word Relation * Response Relation * S-S recall	4.99	7.28	0.69	.493	13.66	7.05	1.94	.053

## Experiment 1b: Results

### Response retrieval effects

According to the same criteria as in Experiment 1a, erroneous probes (6.1% of the trials), probes following erroneous primes (6.2% of the trials) as well as probe RT outliers (4.1% of the trials, leading to a total of 16.4% out of which 0.5% trials have both prime and probe errors, thus resulting in an overall exclusion: 15.9 % of all trials) were removed. *N* = 1 participant was removed from the analysis due to a high error rate (= 30 % errors, with the exclusion criteria being ≥ 25%). Mean probe RT was entered as a dependent variable to a 2 (Response Relation) × 3 (Word Relation) repeated-measures ANOVA.

The results showed a main effect of response relation, reflecting faster performance for RR (*M* = 490 ms) than for RC trials (*M* = 545 ms), *F*(1,128) = 529.08, *p* < .001, η_p_^2^ = 0.81, and a main effect of word relation, F(2,256) = 14.21, *p* < .001, η_p_^2^ = 0.10, due to faster performance in Identical trials (*M* = 512 ms) than Associated and Baseline trials (both *M* = 520 ms). Both effects were qualified by a significant interaction, *F*(2,256) = 109.09, *p* < .001, η_p_^2^ = 0.46, indicating an SRBR effect. For this Experiment, we only preregistered the contrasts that were directly testing the direct and indirect SRBR effects (post hoc contrasts in Experiment 1a). Thus, SRBR effects were computed separately for identical and associated word presentations (see Table 1 for details on effect computation, and Fig. 2). For identical word repetitions, robust SRBR effects emerged that significantly differed from zero, *t*(128) = 12.67, *p* < .001, *d*_*z*_ = 1.11, due to a significant performance benefit of Δ_(B-ID)_ = 26.09 ms, *t*(128) = 12.01, *p* < .001, *d*_*z*_ = 1.05, for RR sequences, and a significant performance cost of Δ_(B-ID)_ = -10.87 ms, *t*(128) = 5.12, *p* = .001, *d*_*z*_ = 0.45, for RC sequences. Similar to Experiment 1a, we conducted a Bayesian one-sample *t*-test to supplement the results and with the alternative hypothesis of predicting an effect significantly higher than 0, we found a BF_+0_ = 3.326×10^+21^ indicating very strong evidence supporting the alternative hypothesis. For associated word presentations in the probe, SRBR effects were absent and did not differ from zero, *t*(128) = 1.03, *p* = .152, *d*_*z*_ = 0.09. The Bayesian analysis revealed a BF_0+_ = 3.6, indicating anecdotal evidence towards the null hypothesis that states that the effect is not significantly greater than zero. These analyses show that the associated probe words did *not* retrieve the responses bound to their associated stimulus.

### Memory of S-S association

Here, the memory of the S-S association was assessed using the performance in the translation questionnaire that was presented at the end of the experiment. For each of the four stimuli from both word pairs, participants were asked what the associated word would be. Mean accuracy rates per item across participants show that participants were able to accurately identify the associated pseudoword word significantly above chance for *dels* (85% correct responses), *t*(128) = 10.78, *p* < .001 (one-tailed), and *mank* (77% correct responses), *t*(128) = 7.16, *p* < .001 (one-tailed). When the equivalent German word was asked, mean accuracy rates also differed significantly from chance, both for *Haus* (81% correct responses), *t*(128) = 9.18, *p* <.001 (one-tailed), and for *Wald* (78% correct responses), *t*(128) = 7.77, *p* <.001 (one-tailed; see Table 2).

This time, the multi-level analysis done to investigate how item-specific memory contributes to indirect retrieval effects for associated words was preregistered. In detail, we ran a linear mixed effect model with random intercept with trial-based predictors as level 1 variables and participants as level 2 predictors and included probe trial RT as dependent variable. We added fixed effects for word relation (only two levels were considered: associated = 0.67 vs. baseline = -0.33, which was contrast coded), response relation (which was contrast coded, RR = 0.5, RC = -0.5), and item-specific accuracy in the cued-recall test for associated words (which was contrast coded accurate = 0.2, inaccurate = -0.8) and their interactions. Participants were added as a random effect. The results are represented in Table 3. The three-way interaction between item-specific S-S recall accuracy, word relation, and response relation missed significance (Table 3). So even in this experiment with multimodal stimulus associations, accurately recalling the associated word did not modulate the strength of the respective indirect prime-response retrieval effects.

## General discussion

Previous research showed that (a) different stimuli that are semantically associated but perceptually dissimilar can also retrieve SR bindings, similar to when the exact stimulus repeats (Frings et al., 2013). Furthermore, (b) stimuli can access and indirectly activate learnt SR contingencies that involve a newly learnt associated stimulus (Arunkumar et al., 2024). Also, (c) transient bindings can form the basis of contingency learning (Giesen et al., 2020; Schmidt et al., 2020). Against this background, we investigated whether retrieval of transient bindings can also be mediated by *newly acquired* S-S associations. We conducted two experiments that used a similar paradigm with the difference being the type of S-S associations used. Participants first learnt novel associations between names and trait adjectives presented visually in Experiment 1a or learnt an association between German words and pseudowords presented audio-visually in Experiment 1b. To test for response retrieval effects, the words used in the prime and probe were either identical, associated, or different. Results are clear-cut and alike in both experiments irrespective of the difference in the type of S-S associations: First, we obtained robust SRBR effects for identical word repetitions in the probe that were in the effect size range that is comparable to other studies on SRBR effects for irrelevant words (e.g., Giesen & Eder, 2022; Giesen & Rothermund, 2011, 2015, 2016) in Experiment 1a and in Experiment 1b (the latter showed even larger SRBR effects). Second and more importantly, SRBR effects were absent for presentations of associated probe words. These results argue against indirect retrieval effects for recently acquired S-S associations. Note that this interpretation is based on null findings from two highly powered, preregistered experiments. Both experiments were sufficiently powered to detect even small effect sizes (*d*_*z*_ = 0.22); furthermore, Bayes factor analyses indicate that the null hypothesis (i.e., absence of SRBR effects for associated words) is 7.5 times more likely than the alternative hypothesis (moderate evidence according to van Doorn et al., 2021) in Experiment 1a and 3.6 times more likely in Experiment 1b. This shows that, in case of transient episodic retrieval, newly learnt associations cannot retrieve responses from associated stimuli.

To support this claim further, we also tested the extent to which the strength of the S-S associations at the level of particular stimuli influenced the presence/absence of an indirect SRBR effect. One might argue that not all participants might have learnt the S-S associations very well. However, in Experiment 1a more than half of all participants had better than chance performance in the memory test (see Footnote 3). An even larger proportion of participants were aware of the S-S associations in Experiment 1b,  which resembled a language-learning scenario and thus possibly made it easier to encode the associations. Moreover, examining the indirect SRBR effects as a function of stimulus-specific recall of the associated stimulus revealed no influence of the memory of S-S association on the SRBR effects. Indirect SRBR effects did not even emerge for stimuli for which the S-S association was correctly recalled. Differences in memory strength for S-S associations alone therefore cannot explain the absence of SRBR effects for associated words.

## Limitations

Possibly, although participants knew which word pairs were presented in the learning phase and could report this knowledge in a later memory test, the words within a pair were not (yet) strongly associated with each other to an extent to which these associations are available for automatic retrieval. Thus, it did not lead to an automatic co-activation of the word that was paired with the retrieval cue. Associations between different stimuli are established in semantic memory over a long time period and due to many pairings, which allow them to be easily accessible to enable retrieval benefits (Chein & Schneider, 2012). This might imply that 40 S1-S2 presentations per each pair as seen in both the studies are (a) sufficient to equip participants with explicit knowledge of which words go together (Arunkumar et al., 2024), but are (b) not sufficient to create new associations in semantic memory to enable for an automatic indirect SR retrieval. Even though language learning tends to store information in semantic long-term memory (Dijsktra & Van Heuven, 2002), our attempt to replicate this with multimodal stimulus pairs containing a German word and pseudoword did not show any indirect retrieval effects. In line with this argument, memory research found that novel word associations need more consolidation, such as a 24-h time period, to show semantic priming effects and elicit automatic retrieval processes (e.g., Bakker et al., 2015). Knowledge of past pairings, then, is not the same as an association, because it cannot trigger episodic retrieval processes. Tentatively, this implies that conceptually mediated retrieval of SR bindings requires the existence of previously established overlearned associations (e.g., Frings et al., 2013). Examining whether participants had only knowledge of pairings but did not yet semantically associate words with each other would require a real test for associations (e.g., a semantic priming paradigm) and might represent a promising avenue for future research.

The present experiments assessed direct and indirect retrieval effects in the same paradigm, yet via different prime-probe sequences: Whereas direct retrieval only operates on probe trials with identical repetition at the stimulus level, indirect retrieval (supposedly) operates on trials in which non-identical, associated stimuli are presented in prime and probe. Effects of direct or indirect retrieval are assessed against baseline trials in which non-identical, non-associated stimuli are presented in prime and probe. We concede that the strong perceptual similarity existing in the measure of direct retrieval (identical stimulus repetition from prime to probe) possibly inflates the size of the direct retrieval effect due to overlap at the semantic *and* perceptual level.^4^ One could even argue that the possibility of retrieval via perceptual similarity might reduce the chances of more indirect retrieval processes (via semantic associations) to come to work. This could potentially be a reason why for the associated words, the indirect retrieval effects were absent. However, this is speculative, and needs to be tested in future studies with an altered design suited to tackle this caveat. Therefore, as an alternative design option for further research, after stimulus-stimulus association learning, the condition of identical stimuli repetition in the probe could be removed, thus presenting only the associated or different stimuli in the prime-probe task. Without identical probes and thus without any interfering retrieval by perceptual similarity, retrieval effects for the associated probes might in fact show. Alternatively, perceptually similar stimulus pairs could be used for both the associated and neutral conditions, in order to test whether indirect retrieval via associations might depend on perceptual similarity. By these modifications, future studies could further test whether newly learnt associations can lead to indirect retrieval.

The present limitations notwithstanding, our study is clearly informative for binding research, because it sheds light on boundary conditions that limit the range of episodic binding and retrieval principles. In line with previous findings (Arunkumar et al., 2022, 2024), it appears that any form of knowledge about stimulus pairings or awareness of contingencies between stimuli and/or responses limits the applicability of episodic accounts like the Binding and Retrieval in Action Control framework (BRAC; Frings et al., 2020) to explain performance.

## Footnotes

1 The pilot experiment (*N* = 71; preregistration can be found here: https://doi.org/10.17605/OSF.IO/ZGDFV) was identical to Experiment 1a, but yielded unexpectedly small SRBR effects with effect sizes for identical stimulus repetitions of *d*_*z*_ = 0.22, which failed the conventional significance criterion (typically, effect sizes for identical word repetitions in color classification tasks produce effect sizes of *d*_*z*_=.35 or higher, cf. Giesen & Eder, 2022; Giesen & Rothermund, 2011, 2015, 2016). Since our study was the first to investigate the possibility of indirect retrieval effects, we settled on the effect size from the pilot study as a more conservative estimate. It was crucial to at least achieve a significant direct retrieval effect to begin with given the modified paradigm, hence the effect size for identical stimuli was used as a reference to determine sample size (Brysbaert, 2019).

2 RT values below 150 ms or higher than 1.5 interquartile range above the 75th percentile of the individual RT distribution were regarded as outliers (Tukey, 1977).

3 In line with our pre-registration, we also looked at the influence of S-S association memory in a person-centered analysis, using participants’ overall accuracy score (aggregated across items). About 56% of our sample had better than chance memory for S-S associations (i.e., a score of 3 or 4). To explore the role of accurate S-S memory for the emergence of indirect retrieval effects, we performed a one-way ANOVA for the five accuracy score groups on SRBR effects for associated words (vs. baseline) with three orthogonal contrasts. Contrast 1 compared participants with scores at or below chance against those with better than chance performance; Contrast 2 compared participants with chance performance to those with below chance performance; Contrast 3 compared participants with a score of 3 versus 4. None of these contrasts was significant, all |*t*| < 1.60, all *p* > .100. Differences in memory strength for S-S associations therefore cannot explain the absence of SRBR effects for associated words.

4 We would like to thank an anonymous reviewer for this perspective.
